# Radiological Growth Rate of Dentigerous Cysts Associated with Mandibular Third Molars: A Retrospective Panoramic Study

**DOI:** 10.3390/medicina62010078

**Published:** 2025-12-30

**Authors:** Ahmet Ferhat Büyükdeniz, Mikail Atabay, Beyza Kaya, Ömer Demir, Hatice Ortaç

**Affiliations:** 1 Department of Oral and Maxillofacial Surgery, Faculty of Dentistry, Dicle University, 21280 Diyarbakir, Turkey; dtmikailatby@gmail.com (M.A.); bkaya@dicle.edu.tr (B.K.); omerdemirdt@gmail.com (Ö.D.); 2 Department of Biostatistics, Institute of Health Sciences, Uludağ University, 16059 Bursa, Turkey

**Keywords:** dentigerous cyst, odontogenic cysts radiography, panoramic, mandibular third molar

## Abstract

*Background and Objectives:* Dentigerous cysts are benign odontogenic lesions associated with the crowns of impacted teeth and are the second most common odontogenic cyst after radicular cysts. Despite being described as slow-growing, their growth has not been numerically quantified. This study aims to determine the radiological growth rate of dentigerous cysts through quantitative analysis, to clarify their biological behavior, improve clinical management, and guide future research by filling a significant gap in the literature. *Materials and Methods:* In this retrospective study, records of 187 patients diagnosed with dentigerous cysts at Dicle University between 2020 and 2024 were reviewed. Twelve patients with cysts associated with mandibular impacted third molars and at least two high-quality panoramic radiographs taken at different time points were included. In all of these patients with adequate and suitable radiographic records, the dentigerous cysts were associated with mandibular third molars, which contributed to the standardization of imaging and measurements. All images were obtained using the same digital system and converted to DICOM format. Maximum anteroposterior (A–P) and inferosuperior (I–S) dimensions were measured twice by the same examiner, and mean values were recorded. Follow-up intervals were noted, and monthly dimensional changes were evaluated. Due to the rarity of untreated cases, a sample of only 12 patients was considered meaningful. *Results:* Most patients were female, with no significant gender differences in age or follow-up time. Cyst dimensions increased over time, showing marked enlargement in the anteroposterior (A–P) direction and a similar upward trend in the inferosuperior (I–S) dimension. *Conclusions:* In our study, growth in the anteroposterior direction was higher than that in the inferosuperior direction, and no significant differences in growth rates were observed with respect to sex or age.

## 1. Introduction

Dentigerous cysts, also referred to as follicular cysts, are benign developmental lesions that arise from the accumulation of fluid between the reduced enamel epithelium and the crown of an unerupted tooth. Dentigerous cysts are most frequently associated with impacted teeth, particularly mandibular third molars. They represent the second most prevalent cystic pathology of the jaws, comprising approximately 24.08–26.6% of all odontogenic cysts [1,2]. Although most frequently diagnosed during the second to fourth decades of life, these cysts can occur across a broad age spectrum. The incidence has been estimated at 1.44 cases per 100 unerupted teeth. While the normal width of dental follicles is approximately 3–4 mm, a follicular space measuring 5 mm or more may provide an indication in favor of a dentigerous cyst. However, this finding should be interpreted in conjunction with other differential diagnoses, including radicular cyst, odontogenic myxoma, odontogenic keratocyst, and unicystic ameloblastoma [3,4]. Mandibular third molars are the most commonly associated teeth, followed by maxillary canines and maxillary third molars [1,5]. Atypical presentations arising from periapical infections of primary teeth have also been reported [6]. Because dentigerous cysts are typically asymptomatic unless secondarily infected, they are often identified incidentally during routine dental radiographic evaluations [7,8].

Histological sections of dentigerous cysts show hyperplastic, non-keratinized stratified squamous epithelium accompanied by fibrous connective tissue, prominent rete ridges, and an infiltrate of inflammatory cells [9]. Radiographically, dentigerous cysts present as well-defined, unilocular radiolucent lesions encircling the crown of an unerupted tooth, typically outlined by a sclerotic border. These cysts exhibit a tendency for progressive enlargement, and the pressure exerted on adjacent teeth may result in tooth displacement and localized bone resorption. Depending on the extent of osseous resorption, the weakened bone although rarely may become prone to iatrogenic fracture due to compromised structural integrity [10]. As the cyst continues to enlarge, it may lead to progressive displacement of adjacent teeth, paresthesia in the inferior alveolar nerve in some cases, and devitalization of neighboring teeth [11].

A definitive diagnosis requires a comprehensive evaluation integrating clinical findings with radiographic and histopathological analyses. Cone-beam computed tomography (CBCT) has emerged as a valuable modality for precise preoperative assessment and surgical planning. However, in our study, due to the unavailability of historical CBCT images for the patients, the analyses were conducted using panoramic radiographs. Treatment modalities include decompression to reduce cystic size, enucleation, resection, or a combination of these techniques [10,12]. Early and accurate identification of the lesion is essential for selecting the optimal therapeutic approach [13].

Information regarding the radiological growth rate of dentigerous cysts remains limited, and most existing studies rely primarily on qualitative descriptions. This limitation complicates the objective determination of follow-up intervals and the assessment of optimal timing for surgical intervention. The lack of quantitative data therefore introduces uncertainty into clinical decision-making processes.

The aim of this study was to quantitatively assess the radiographic growth rate of dentigerous cysts. The findings may contribute to a clearer understanding of the growth behavior of these lesions. Quantitative measurements may enable estimation of the average monthly growth rate. This information may assist in defining appropriate follow-up intervals. It may also help identify lesions with relatively rapid progression. The results may support more informed decision-making in surgical planning. In addition, this study aims to provide a comparable dataset that may serve as a reference for future studies in this field.

## 2. Materials and Methods

### 2.1. Ethical Considerations

This study was carried out using retrospective radiographic records from 12 patients who presented to the Faculty of Dentistry at Dicle University. Ethical approval was obtained from the Dicle University Faculty of Dentistry Clinical Research Ethics Committee (Protocol Code: 2025-53, Date: 25 June 2025), and the study was conducted in accordance with the principles of the Declaration of Helsinki.

### 2.2. Data Collection and Study Design

**Figure 1:**
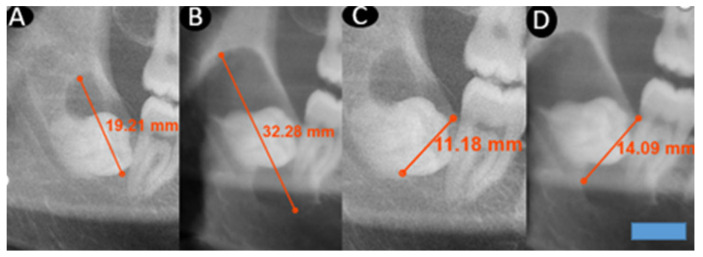
This figure demonstrates changes in cyst dimensions based on calibrated linear measurements obtained from panoramic radiographs at baseline and at long-term follow-up. The calibrated measurements highlight progressive enlargement over time and provide visual corroboration for the calculated growth rates derived from radiographic length assessments. A blue scale bar with a length of 10 mm and a height of 3 mm was added in the lower right corner of the image. (**A**): A–P first measurement. (**B**): A–P second measurement after 64 months. (**C**): I–S first measurement. (**D**): I–S second measurement after 64 months.

**Figure 2:**
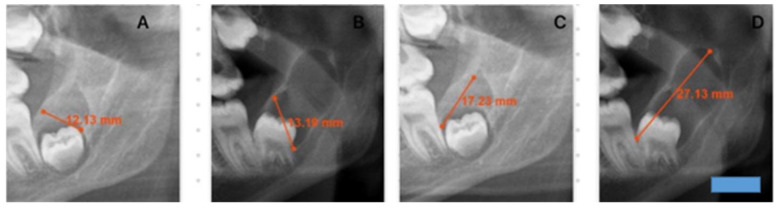
This figure demonstrates changes in cyst dimensions based on calibrated linear measurements obtained from panoramic radiographs at baseline and at long-term follow-up. The calibrated measurements highlight progressive enlargement over time and provide visual corroboration for the calculated growth rates derived from radiographic length assessments. A blue scale bar with a length of 10 mm and a height of 3 mm was added in the lower right corner of the image. (**A**): I–S first measurement. (**B**): I–S second measurement after 36 months. (**C**): A–P first measurement. (**D**): A–P second measurement after 36 months.

This retrospective study evaluated the radiographic records of patients who presented to the Department of Oral and Maxillofacial Surgery at Dicle University Faculty of Dentistry between 2020 and 2024, underwent biopsy, and received a histopathological diagnosis of dentigerous cysts. Among 187 patients with a definitive post-treatment diagnosis, 12 had previously been scheduled for surgical intervention but failed to attend their appointments (Table 1). Only histopathologically confirmed dentigerous cysts were included in the study. Consequently, radiolucent lesions that could mimic dentigerous cysts were excluded, which inherently limited the scope for comparative analysis. These individuals later returned to the clinic after varying intervals of time. Prior to treatment, new panoramic radiographs were obtained, and repeat biopsies reconfirmed the diagnosis of dentigerous cysts. As patients were informed about the potential risk of neoplastic transformation of these cysts, they were encouraged not to delay treatment, and due to the small number of individuals who postponed their procedures, the sample was limited to 12 patients. To be included in the study, lesions had to remain untreated solely for patient-related reasons, and follow-up panoramic radiographs suitable for comparison had to be available at least six weeks after the initial imaging. In our study, among the included radiographs, the earliest follow-up visit occurred 8 months after the initial radiograph, whereas the latest was recorded 108 months after the initial radiograph. For these 12 patients, the initial panoramic radiographs were compared with follow-up images obtained several months later, focusing specifically on lesions involving the same impacted teeth and corresponding anatomical regions (Figure 1 and Figure 2). Dimensional changes in the cysts over time were measured, and monthly growth rates were subjected to statistical analysis. Only high-quality panoramic radiographs that were clear, properly positioned, and minimally affected by patient-related factors such as movement or improper alignment were included. All images were acquired using devices of the same make, model, and system (Planmeca ProMax 2D (Planmeca Oy, Helsinki, Finland) with DentAssist software (version 4.1.200)), ensuring imaging standardization. The original radiographs were subsequently converted to DICOM format to enable precise digital analysis, and all radiographic measurements were calibrated using the built-in calibration system integrated into the imaging device. In this study, dentigerous cyst lesions were identified on panoramic radiographs, and their dimensions were measured to evaluate changes over time. Because lesion growth rates may differ according to anatomical location, only dentigerous cysts associated with impacted mandibular third molars were included. For each patient, the largest anteroposterior (A–P) and inferosuperior (I–S) dimensions of the cyst were recorded on two panoramic radiographs obtained at different time points. Each dimension was measured twice on every radiograph, and the mean value of the two measurements was used for comparison. All measurements were performed by the same investigator, with each measurement repeated twice to enhance consistency and minimize intra-observer variability. The time intervals between radiographs were standardized and expressed in months for statistical analysis. The monthly growth rate represents a mathematical standardization obtained by dividing the dimensional difference between two radiographs by the elapsed time; it is not intended to directly observe short-term radiological changes.

The study was restricted to mandibular third molars to maintain anatomical consistency and minimize variability related to differing eruption paths, angulations, and radiographic distortion among other tooth types. Although dentigerous cysts can involve other teeth, the retrospective archive did not contain a sufficient number of non–third molar cases with serial high-quality panoramic radiographs for meaningful comparison.

### 2.3. Intraclass Correlation Coefficient

In this study, Intraclass Correlation Coefficient (ICC) analysis was conducted to evaluate the consistency of measurements performed by the same observer at two separate time points. The repeated measurements were conducted two days apart to minimize recall bias and to ensure an independent, unbiased reassessment by the same examiner. Using a two-way mixed-effects model with an absolute-agreement definition, the single-measure ICC was calculated as 0.388 (95% CI: −0.103 to 0.786), indicating low intra-observer reliability for individual measurements. The average-measure ICC was 0.559 (95% CI: −0.229 to 0.880), reflecting a moderate level of agreement when the mean of repeated measurements was considered. Additionally, the Cronbach’s alpha coefficient was 0.839, demonstrating generally good internal consistency across the measurement set.

For the inferosuperior measurements, intra-observer reliability was likewise assessed using ICC analysis. The single-measure ICC, calculated using a two-way mixed-effects model with absolute agreement, was 0.228 (95% CI: −0.122 to 0.637), indicating poor reliability at the individual measurement level. The average-measure ICC was 0.372 (95% CI: −0.278 to 0.778), reflecting low agreement even when mean values were considered. The Cronbach’s alpha coefficient was 0.626, representing a borderline acceptable level of internal consistency.

### 2.4. Statistical Analysis

Duration (months), A–P Measurement 1, A–P Measurement 2, I–S Measurement 1, and I–S Measurement 2 variables were assessed for normality using the Shapiro–Wilk test. Descriptive statistics were expressed as mean ± standard deviation and median (IQR). Categorical variables were summarized as n (%). In comparisons between two groups, the Independent samples *t*-test was used when data followed a normal distribution. Paired sample *t*-tests were performed to compare pre- and post-measurement values when the assumption of normality was satisfied.

To assess intra-observer reliability for repeated measurements, the Intraclass Correlation Coefficient (ICC) was calculated using a two-way mixed-effects model with an absolute-agreement definition (single measures). All statistical analyses were performed using SPSS software (IBM Corp. Released 2017. IBM SPSS Statistics for Windows, Version 25.0, Armonk, NY, USA), and the Type I error level was set at 5%.

## 3. Results

On the panoramic radiographs of the selected patients, pathological lesions were evaluated in two dimensions: anteroposterior (A–P) and inferosuperior (I–S). For each lesion, the longest measurable diameter in both directions was recorded. The relationships between lesion dimensions and variables such as patient age, sex, and the interval before treatment were then analyzed. All collected data were organized and presented in tabular format (Table 2).

This table presents each patient’s age, sex, and the anatomical location of the cyst, with the number of the associated impacted tooth indicated according to the FDI num- bering system. It includes the anteroposterior (A–P) and inferosuperior (I–S) dimensions measured at the initial assessment, as well as the corresponding A–P and I–S values obtained during the second evaluation. The time interval between the two assessments is also provided in months. This comprehensive tabular layout allows readers to clearly visualize the longitudinal dimensional changes in the cysts for each individual patient.

The mean age of the patients was 25.58 ± 9.16 years. In terms of sex distribution, 58.3% of the participants were female (n = 7) and 41.7% were male (n = 5). The mean follow-up duration was 48.5 ± 31.92 months. The mean initial anteroposterior (A–P) cyst dimension measured 13.5 ± 4.29 mm, increasing to 21.92 ± 7.83 mm at the second evaluation. The mean initial inferosuperior (I–S) dimension was 10.75 ± 1.96 mm, increasing to 15.75 ± 4.49 mm at the second evaluation.

The mean age of female patients was 23.43 ± 7.14 years, whereas the mean age of male patients was 33 ± 11.61 years; however, this difference was not statistically significant (*p* = 0.359). The mean follow-up duration was 47 ± 32.27 months for female patients and 50.6 ± 35.09 months for male patients, with no significant difference between the groups in terms of follow-up time (*p* = 0.858).

The mean initial A–P measurement was 12.86 ± 4.59 mm in female patients and 14.4 ± 4.16 mm in male patients, with no statistically significant difference between the groups observed (*p* = 0.565). At follow-up, the mean A–P measurement was 22.29 ± 8.79 mm in females and 21.4 ± 7.23 mm in males, again demonstrating no significant difference (*p* = 0.857). The mean initial I–S measurement was 10.86 ± 2.48 mm in females and 10.6 ± 1.14 mm in males, with no significant group difference (*p* = 0.835). The mean I–S dimension at the second time point was 16.57 ± 5.88 mm in females and 14.6 ± 0.89 mm in males, with no significant group difference (*p* = 0.415) (Table 3).

The mean anteroposterior (A–P) diameter of the cyst measured 13.5 ± 4.29 mm at the initial evaluation and increased to 21.92 ± 7.83 mm at the follow-up, demonstrating a statistically significant enlargement of the lesion’s longest dimension (*p* < 0.001). Similarly, the mean inferosuperior (I–S) diameter increased from 10.75 ± 1.96 mm at the first measurement to 15.75 ± 4.49 mm at the second, indicating a significant increase in the cyst’s shorter dimension over time (*p* = 0.001).

### 3.1. Monthly Growth Rate of the Cyst:

**Figure 3:**
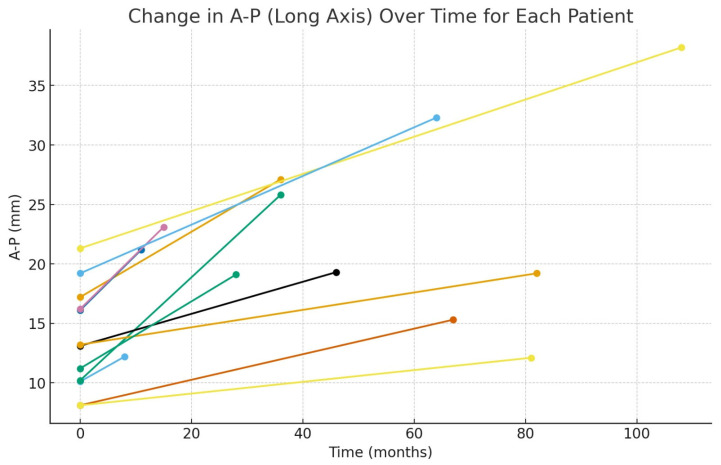
Graph showing the monthly changes in the anteroposterior growth of the cyst. The graph displays changes in anteroposterior (A–P) length on the y-axis and the elapsed time on the x-axis. Measurements obtained from the initial and final panoramic radiographs of twelve patients are plotted as individual points. To illustrate the time-dependent growth trend, the two measurement points corresponding to each patient were connected with a linear segment. Each line, distinguished by a different color, represents the individual growth trajectory of a single patient.

**Figure 4:**
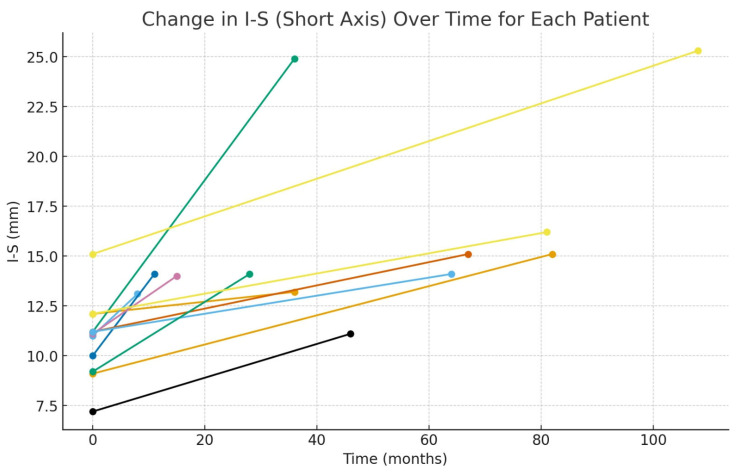
Graph showing the monthly changes in the inferosuperior growth of the cyst. The graph displays changes in inferior–superior (I–S) length on the y-axis and the elapsed time on the x-axis. Measurements obtained from the initial and final panoramic radiographs of twelve patients are plotted as individual points. To illustrate the time-dependent growth trend, the two measurement points corresponding to each patient were connected with a linear segment. Each line, distinguished by a different color, represents the individual growth trajectory of a single patient.

**Figure 5:**
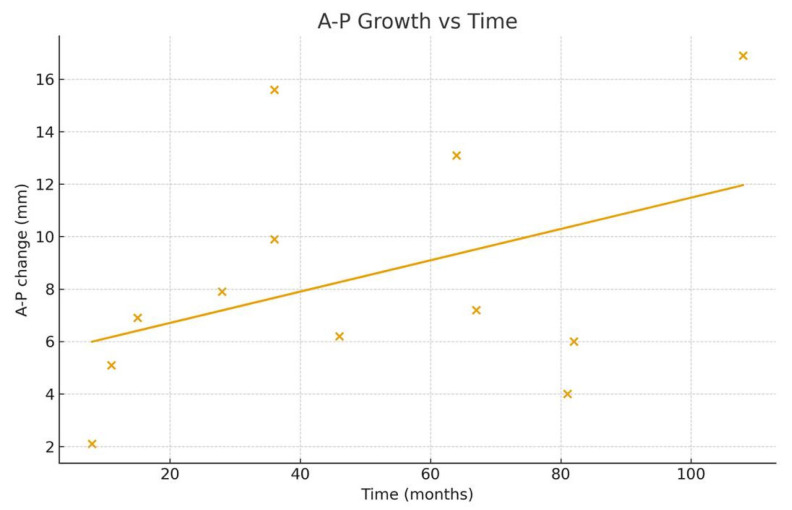
This figure illustrates the relationship between elapsed time (months) and the anteroposterior (A–P) dimensional change in dentigerous cysts. Each point represents the net A–P change (AP2–AP1) derived from two serial panoramic radiographs of the same patient. A positive value indicates an increase in the long axis dimension between the first and second imaging sessions. A simple linear regression line is superimposed to demonstrate the overall growth trend across individuals.

**Figure 6:**
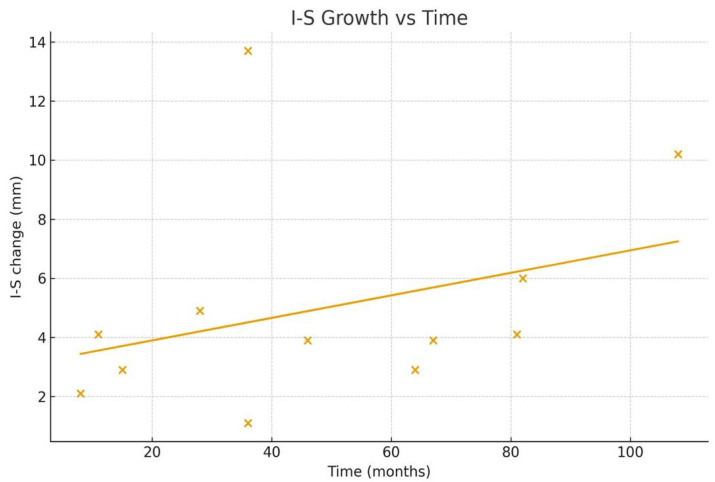
This figure shows the association between elapsed time (months) and the inferosuperior (I–S) dimensional change in dentigerous cysts. Each scatter point corresponds to the net I–S difference (IS2–IS1) measured between two sequential panoramic radiographs. The fitted regression line reflects the general pattern of I–S enlargement over the observation period.

The monthly growth rate of the cyst was calculated as 0.24 ± 1.15 (0.15–0.34) mm for the anteroposterior (A–P) diameter and 0.15 ± 0.13 (0.07–0.23) mm for the inferosuperior (I–S) diameter. These findings indicate a progressive increase in cyst size over time, with a more pronounced growth pattern observed in the A–P dimension (Figure 3, Figure 4, Figure 5 and Figure 6).

### 3.2. Monthly Percentage Growth Rate of the Cyst:

The monthly percentage growth rate of the cyst was also evaluated, revealing an average increase of 1.85% ± 1.20% (1.09–2.62) in the anteroposterior (A–P) diameter and 1.46% ± 1.19% (0.69–2.22) in the inferosuperior (I–S) diameter. These values reflect the relative monthly expansion of the cyst dimensions.

### 3.3. Monthly Increase in Cyst Diameter by Sex:

In female patients, the average monthly growth rate of the cyst in the anteroposterior (A–P) dimension was 0.24 ± 0.12 (0.13–0.34) mm, whereas in male patients it was 0.25 ± 0.20 (0–0.49) mm. Similarly, the monthly growth rate in the inferosuperior (I–S) dimension was 0.16 ± 0.13 (0.04–0.27) mm for females and 0.15 ± 0.14 (0–0.32) mm for males. These findings demonstrate that cyst growth rates were comparable between sexes, with no meaningful differences observed.

## 4. Discussion

In the present study, 7 female and 5 male patients met the inclusion criteria. Although prior studies have reported a higher incidence of dentigerous cysts in males [14,15]. The predominance of female patients in our sample may be related to the limited sample size of the study.

Dentigerous cysts are most commonly associated with impacted mandibular third molars, followed by impacted maxillary canines [16]. In our study, all cysts were linked to impacted mandibular third molars.

These lesions are benign, slow-growing developmental odontogenic cysts and are most frequently diagnosed during the second to third of life [17]. Consistent with the existing literature, 75% of the patients in our study were in the second to fourth decades of life.

These lesions characteristically present as solitary and clinically silent entities and are most often identified incidentally during routine radiographic examinations. Numerous studies in the literature have demonstrated that dentigerous cysts typically exhibit slow growth [18]. In the present study, the monthly percentage growth rate of these cysts was assessed, revealing an average increase of 1.85% ± 1.20% in the anteroposterior (A–P) diameter and 1.46% ± 1.19% in the inferosuperior (I–S) diameter.

Dentigerous cysts possess the potential for neoplastic transformation and, if left untreated, may progress to squamous cell carcinoma [19]. For this reason, lesions suspected to be dentigerous cysts should be managed as early as possible. In our study, although patients were initially scheduled for surgical intervention at the time of diagnosis, they did not attend their appointments as planned. These patients later returned to our faculty, at which point their cysts were treated. Only individuals whose biopsy results confirmed a definitive diagnosis of dentigerous cyst were included in the study. Final diagnoses were established through histopathological examination of the surgical specimens.

Dentigerous cysts may also develop from follicular cysts [20]. In this study, we examined cysts associated with impacted third molars that were histopathologically confirmed as dentigerous cysts. Initial radiographic assessments were considered provisional diagnoses, while definitive confirmation was established through histopathological analysis of biopsy specimens obtained during treatment following the second radiograph. It should be noted that only a provisional diagnosis can be made from the initial radiographs, reflecting a scientific limitation; histopathology remains the gold standard. Even if some of the cysts included in our study may have originated from follicular cysts, quantifying their growth rates using objective numerical data provides clinically and scientifically valuable information.

Cone-beam computed tomography (CBCT) provides more detailed and diagnostically valuable imaging of jaw lesions than panoramic radiography [21]. In the present study, however, because historical CBCT data were not available for the included patients, all analyses were conducted using panoramic radiographs. Conducting a similar study with CBCT would undoubtedly enhance the scientific strength and diagnostic accuracy of the findings. Nevertheless, the limited number of patients who missed their initially scheduled treatments, combined with the difficulty of accessing past CBCT records, restricts the feasibility of evaluating cyst growth rates using CBCT in this context.

In our study, the sample size was limited to 12 patients, and more robust conclusions could be drawn from a larger dataset. Following initial diagnosis and counseling, most patients comply with treatment recommendations due to the potential risk of neoplastic transformation; therefore, the number of individuals who failed to attend their scheduled treatments was inherently small. As a result, future research aiming to analyze cyst growth with a larger sample should consider multicenter study designs involving multiple oral and maxillofacial surgery clinics. Such an approach would facilitate the inclusion of a broader patient population. We believe that the findings of our study offer valuable preliminary insights that may guide and support future investigations in this field.

Odontogenic cysts may demonstrate varying growth patterns depending on their anatomical location [22]. In our study, all lesions were dentigerous cysts associated with impacted mandibular third molars. Future investigations examining the growth rates of dentigerous cysts in different anatomical regions could yield more comprehensive and region-specific data.

In our retrospective dataset, the limited availability of serial radiographs for dentigerous cysts involving teeth other than mandibular third molars precluded comparative analyses across different anatomical sites. Consequently, larger, multicenter studies with more diverse patient cohorts are warranted to robustly evaluate potential variations in growth rates among dentigerous cysts associated with different teeth and to enhance the generalizability of the findings.

It is important to note that this study exclusively included patients who missed their scheduled surgical appointments. As a result, the sample may not be fully representative of the broader dentigerous cyst population. Specifically, individuals in this subgroup may systematically differ with respect to health behaviors, symptom perception, and access to healthcare services. Consequently, the generalizability of the findings may be limited. This issue should therefore be considered not merely as a logistical constraint but also as a potential source of selection bias, which warrants careful consideration when interpreting the results.

### 4.1. Strengths

One of the strengths of our study is that it provides quantitative data on the time intervals during which dentigerous cysts may reach potentially hazardous sizes, which is particularly relevant in the management of urgent medical conditions or trauma cases. Furthermore, the findings offer a foundation for future research aimed at improving the understanding of the biological behavior and growth dynamics of these cysts.

### 4.2. Limitations

- Achieving an adequate sample size is challenging because patients with cystic lesions such as dentigerous cysts given their potential risk for neoplastic transformation are unlikely to delay or miss their scheduled surgical appointments.
- Panoramic radiographs may exhibit distortions depending on head and neck positioning. Although minimal distortion was observed in our patients, panoramic imaging does not provide the same level of precision as advanced imaging modalities such as CBCT.
- While the diagnosis of dentigerous cysts could be confirmed on the second panoramic radiographs obtained at the time of treatment, the initial panoramic images taken before patients returned for surgery could only provide a provisional diagnosis. This limitation arises because dentigerous cysts may also originate from dental follicles. Nevertheless, this constraint does not diminish the value of the study and still permits the extraction of clinically relevant and predictive insights for follow-up.
- Measurement reliability constitutes a notable limitation of the present study. The intraclass correlation coefficients demonstrated low to moderate agreement, particularly for single measurements, which may compromise the precision of small monthly growth estimates. Because the observed dimensional changes frequently occurred at submillimetric levels, a portion of the measured variation may fall within the range of measurement error. Furthermore, the absence of inter-observer reliability assessment limits the reproducibility of the findings across different observers and centers. Accordingly, the reported growth rates should be interpreted with caution and considered preliminary reference values rather than definitive quantitative measurements.
- Another limitation of this study is that the examiner was not fully blinded to the chronological sequence of the radiographs. Awareness of whether an image represented the baseline or follow-up stage may have introduced measurement bias. To mitigate this, all radiographs were randomized before each measurement session, and the second set of measurements performed two days later was conducted using an independently re-randomized order. This procedure was designed to minimize potential unconscious bias to the greatest extent possible.

## 5. Conclusions

In our study, the monthly percentage growth rate of dentigerous cysts was evaluated, demonstrating an average increase of 1.85 ± 1.20% in the anteroposterior (A–P) diameter and 1.46 ± 1.19% in the inferosuperior (I–S) diameter. Additionally, the mean monthly linear growth rate was 0.24 ± 1.15 mm for the A–P dimension and 0.15 ± 0.13 mm for the I–S dimension. No significant differences in monthly growth rates were observed with respect to sex or age.

## Figures and Tables

**Table 1:** Patient Selection Summary.

Criterion	Number of Patients
Total number of patients with a biopsy-confirmed diagnosis of dentigerous cyst	187
Number of patients with dentigerous cysts associated with mandibular third molars	153
Number of patients who had at least two panoramic radiographs taken at different time points in the retrospective dataset (patients who initially underwent imaging but did not receive treatment and later returned after a prolonged interval for repeat radiographic evaluation)	17
Number of patients whose two panoramic radiographs were deemed suitable with respect to distortion and were therefore included in the study	12

**Table 2:** Individual Patient Characteristics and cyst Measurements.

Patient ID	Sex	Age (Years)	Location	Time (Months)	A–P 1 (mm) Long Axis	A–P 2 (mm) Long Axis	I–S 1 (mm) Short Axis	I–S 2 (mm) Short Axis
1	Female	17	38	36	17.2	27.1	12.1	13.2
2	Female	28	48	8	10.1	12.2	11	13.1
3	Female	21	38	36	10.2	25.8	11.2	24.9
4	Female	32	38	108	21.3	38.2	15.1	25.3
5	Male	11	38	11	16.1	21.2	10	14.1
6	Female	12	38	67	8.1	15.3	11.2	15.1
7	Male	23	38	15	16.2	23.1	11.1	14
8	Female	25	38	46	13.1	19.3	7.2	11.1
9	Male	37	38	82	13.2	19.2	9.1	15.1
10	Male	39	48	64	19.2	32.3	11.2	14.1
11	Female	29	38	28	11.2	19.1	9.2	14.1
12	Male	33	38	81	8.1	12.1	12.1	16.2

**Table 3:** Radiological measurements and growth rate data of dentigerous cysts.

	Total	Female (n = 7)	Male (n = 5)	*p*-Value
Age (years)	25.58 ± 9.16	23.43 ± 7.14	33 ± 11.61	0.359 ^a^
	26.5 (15)	25 (12)	33 (21)	
Duration (months)	48.5 ± 31.92	47 ± 32.27	50.6 ± 35.09	0.858 ^a^
	41 (59)	36 (39)	64 (69)	
A–P Measurement 1	13.5 ± 4.29	12.86 ± 4.59	14.4 ± 4.16	0.565 ^a^
	13 (7)	11 (7)	16 (7)	
A–P Measurement 2	21.92 ± 7.83	22.29 ± 8.79	21.4 ± 7.23	0.857 ^a^
	20 (11)	19 (12)	21 (12)	
I–S Measurement 1	10.75 ± 1.96	10.86 ± 2.48	10.6 ± 1.14	0.835 ^a^
	11 (3)	11 (3)	11 (2)	
I–S Measurement 2	15.75 ± 4.49	16.57 ± 5.88	14.6 ± 0.89	0.415 ^a^
	14 (3)	14 (12)	14 (2)	

Data are presented as mean ± standard deviation and median(IQR). ^a^: Independent samples *t*-test.

## Data Availability

The original contributions presented in this study are contained within the article. The underlying data supporting the findings of this study are available from the corresponding author upon reasonable request. Further inquiries may be directed to the corresponding author.

## Abbreviations

The following abbreviations are used in this manuscript:

CBCT	Cone Beam Computed Tomography
DICOM	Digital Imaging and Communications in Medicine
ICC	Intraclass Correlation Coefficient
A–P	Anteroposterior
I–S	Inferosuperior
